# Measuring the degree of integration for an integrated service network

**DOI:** 10.5334/ijic.835

**Published:** 2012-09-18

**Authors:** Chenglin Ye, Gina Browne, Valerie S Grdisa, Joseph Beyene, Lehana Thabane

**Affiliations:** Department of Clinical Epidemiology and Biostatistics, McMaster University, Hamilton ON, L8S 4L8 Canada; School of Nursing, McMaster University, Hamilton ON, L8S 4L8 Canada; Healthcare Advisory, Management Consulting, KPMG Canada, Toronto ON, Canada; Department of Clinical Epidemiology and Biostatistics, McMaster University, Hamilton ON, L8S 4L8 Canada; Department of Clinical Epidemiology and Biostatistics, McMaster University, Hamilton ON, L8S 4L8 Canada

**Keywords:** integration measure, perception, expectation, collaboration agreement

## Abstract

**Background:**

Integration involves the coordination of services provided by autonomous agencies and improves the organization and delivery of multiple services for target patients. Current measures generally do not distinguish between agencies’ perception and expectation. We propose a method for quantifying the agencies’ service integration. Using the data from the Children’s Treatment Network (CTN), we aimed to measure the degree of integration for the CTN agencies in York and Simcoe.

**Theory and methods:**

We quantified the integration by the agreement between perceived and expected levels of involvement and calculated four scores from different perspectives for each agency. We used the average score to measure the global network integration and examined the sensitivity of the global score.

**Results:**

Most agencies’ integration scores were <65%. As measured by the agreement between every other agency’s perception and expectation, the overall integration of CTN in Simcoe and York was 44% (95% CI: 39%–49%) and 52% (95% CI: 48%–56%), respectively. The sensitivity analysis showed that the global scores were robust.

**Conclusion:**

Our method extends existing measures of integration and possesses a good extent of validity. We can also apply the method in monitoring improvement and linking integration with other outcomes.

## Background

Spending on healthcare in Canada continues to outpace government revenue and economic growth [1]. In 2010, the total Canadian healthcare expenditures were approximately $191.6 billion [2]. Fraser Institute, a Canadian think-tank, forecasts that if the recent trend continues, six of 10 provincial governments will spend more than half of total revenue on healthcare by the year 2020 [3]. Many factors contribute to escalating healthcare costs that include: development of new drugs, availability of expensive health services, and an increase in co-morbid chronic conditions in our aging populations [4–6]. The sustainability of the existing Canadian universal care system is a growing concern [7].

In general, patients with chronic illness or functional limitations are the major consumers of community health services [8, 9]. For example, in addition to taking psychopharmacologic drugs, patients with mental illness would need psychiatric counseling, rehabilitative therapy, and other health and social services to help them and their families. Usually, such primary care services are delivered by community health centres—which are operated autonomously [10]. Patients often receive help for a single problem at a time and endure duplicative processes to receive multiple services. With limited resources, we can hardly meet the total needs of a population by providing health services in an isolated and fragmented fashion. As a result, patients may experience prolonged wait-times and courses of treatment.

Over the last decade, integration has been advocated as a viable strategy for improving the organization and delivery of health services. A majority of the literature on integrating services discusses the integration of health services on primary care, hospital care, or mental health care [8–11]. Often, the integration of health services is framed under the notion of ‘continuity of care’ [12]. A prominent hypothesis is that integration of health services leads to higher quality of care at a lower cost and maintains or improves patients’ health and satisfaction [13, 14]. In this paper, we adopt the definition introduced by Browne et al., who define integration as the coordination of a comprehensive spectrum of services (e.g., health, education, community and social services) provided by multiple agencies [10]. We use providers and agencies interchangeably to represent the publicly funded organizations that deliver community health services.

The integrated approach of delivering services has potential advantages. Patients will receive comprehensive care and are likely to gain a much better outcome. Another advantage is that service providers can improve their caseload by coordinating with other providers and reducing duplication, and thus, better meet the overall needs of a population. There are different ways to initiating integration within a community. One is by legislative change, such as the Health Action Zones in the United Kingdom whereby a whole geographic area receives the total funding for public and health services. The implementation of Local Health Integration Networks (LHINs) in Ontario, Canada is another example where local region receives funds for acute, community, and long-term care services. Regional health authorities develop service agreements with performance indicators. Savings are kept within the region and used for other priorities. The other approach begins at a local level whereby agencies collaborate together to serve more clients without increasing funding allocation. The local community develops own collaboration in service delivery. The Children’s Treatment Network (CTN) in Ontario, Canada is an example of the third approach, for serving the children with complex health problems.

Studies have shown that integrating local services improves patient outcomes, such as reducing functional decline in the elderly [15], preventing avoidable hospitalizations [16], and minimizing the risk of developing diabetes-related complications [17]. One sentence summarizes the current evidence: healthcare providers can achieve target outcomes more easily with less investment by coordinating available, necessary, and preferable human services to patients [18]. From a societal perspective, proactive and comprehensive services are more effective and less expensive because giving people what they need in a coordinated fashion results in a reduced use of other services [19]. The emphasis on human services is the result of accumulating evidence that the factors (besides genetic predisposition) determining health are social, environmental, educational, and personal in nature. However, the extent to which those services are integrated for addressing patients’ health needs is often unknown and rarely measured.

Without quantifying the degree of integration, the effect of collaborative effort can be hardly identified. Without measuring and monitoring the actual integration, it is difficult for network planners to make decisions and confirm successful implementation [20]. Thus, we need a valid method for measuring the degree to which a network has achieved in integrating services. A recent systematic review [13] identified 18 quantitative measures of integration. None of those measures separate agencies’ current perception of involvement from their expectation. Since agencies would differ in the level of involvement necessary to meet patients’ needs, a rational measure should reflect the agreement between perceived and expected levels of involvement. In marketing, researchers have used the gap between the expected performance and the perceived experience as an objective measure of customer satisfaction [21]. Current measures generally do not distinguish between agencies’ perception and expectation on integration although separate measures offer an appropriate and reliable way to identify the ‘gap’ in integration [22]. Browne et al. develop the Human Service Integration Measure (HSIM) scale [10] that includes separate measures of observed and expected integrations. Another key limitation in existing measures is the common assumption that a higher level of involvement is always a better integration. A high level of input by an agency is not necessarily an agreed-upon involvement by all agencies [10, 13, 23]. A measure of integration needs to account for the expectations of all underlying agencies in their pursuit of a common goal to improve patient outcomes [24–26]. In this paper, we introduce a new method to measure the degree of service integration among agencies. Our proposed method has unique strengths by: quantifying the gap between perceived and expected levels of involvement within a network; creating a relational score by the agreement among all agencies; measuring the integration from four different perspectives; and creating integration scores for individual agencies and global integration scores for the entire network as a whole. In this paper, we use the words ‘perceived’, ‘observed’, and ‘actual’ interchangeably to mean the current level of involvement perceived by agencies. We use the words ‘expected’ or ‘optimal’ to mean the level of involvement expected by agencies. Using the data from the Children’s Treatment Network (CTN), our objectives are to: 1) measure the degree of integration among agencies of CTN; 2) calculate global integration scores of CTN; and 3) assess the sensitivity of the global integration score based on different approaches for estimating the global integration score.

## Theory and methods

### Study design and study population

The data were drawn from a cross-sectional study that evaluated the integration of CTN in 2006, one year after its inception. The study measured the degree of integration among agencies of CTN in Simcoe and York separately.

In Simcoe County and York Region of Ontario, Canada, families with severely disabled child(ren) had limited access to specialized treatments and had to travel outside their region to receive services. They used services in a disjointed and self-directed manner and often received suboptimal outcomes. In response to these problems, Simcoe County and York Region launched the CTN in 2005. Targeting the children with complex health problems, local service agencies in CTN collaborated together to deliver comprehensive therapeutic and psychosocial services to the children and their families. This innovative and proactive model integrated existing service agencies—including health, recreational, educational, social, mental health, and community resources (www.ctn-simcoeyork.ca). Each family served by CTN had a unique team of service providers for a long-term basis. The interdisciplinary team provided a single point of contact, health assessment, service coordination, and a comprehensive plan of care for the children.

### Characteristics of the CTN agencies

At the time of measurement, there were 27 and 36 agencies in Simcoe and York, respectively. The CTN represented health, educational, social, justice, recreational, and cultural sectors. There were different types of agencies in each sector that included: early years, Healthy Babies, adolescent support, rehabilitation, home care, social assistance, child protection, mental health, recreation, leisure services, etc.

### The Human Service Integration Measure Scale

**Figure 1.**
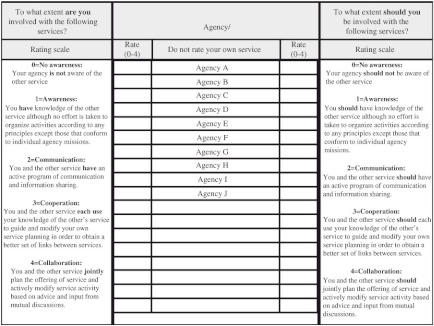
The Human Service Integration Measure Scale.

To quantify the level of partnership involvement, we used the latest version of the Human Service Integration Measure (HSIM) scale, a 5-point ordinal scale developed by Browne et al., to validate their integration framework [10]. Representatives of agencies can fill out the measures by web-form, phone, or in-person interview. An interviewer will ask each agency to rate its current and expected levels of involvement with the other agencies within the network. An example of the measure is provided in Figure 1. For agencies that have more than one representative, the interviewer will average the ratings from all representatives and round it to the nearest integer.

### Organizing the data for calculating integration

**Figure 2.**
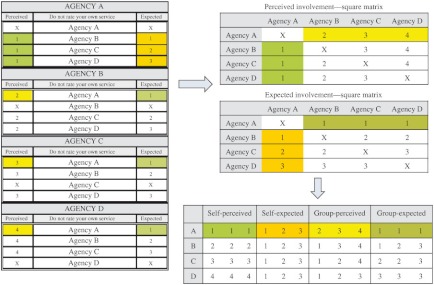
An example for organizing the data.

We have developed two square matrices to organize the responses of perceived involvement and expected involvement, respectively. Each column in the matrix contains an agency’s ratings on every other agency (i.e., self-ratings) and each row contains other agencies’ ratings on the same agency (i.e., group-ratings). An illustrative example is provided in Figure 2. In the matrix of perceived involvement, the first column contains the level of involvement that Agency A has perceived with every other agency (e.g., 1, 1, and 1) and the first row contains the level of involvement that other agencies have perceived with Agency A (e.g., 2, 3 and 4). Similarly, in the matrix of expected involvement, the first column contains the level of involvement that Agency A has expected with every other agency (e.g., 1, 2, and 3) and the first row contains the level of involvement that other agencies have expected with Agency A (e.g., 1, 1, and 1). The ratings for other agencies are organized in the same way. Every agency has four types of ratings, namely, self-perceived, self-expected, group-perceived, and group-expected ratings.

### Calculating the agency integration scores

**Figure 3.**
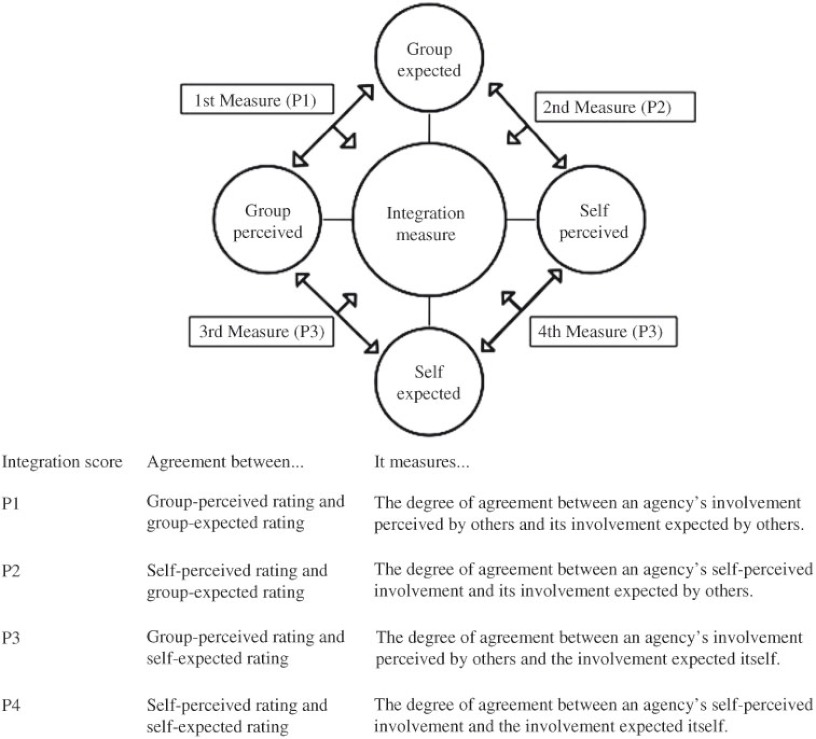
The framework of measuring integration.

We measured an agency’s integration score by the agreement between perceived and expected involvements with other agencies and defined the agreement as the percentage of pairs of agreed perceived and expected ratings. Thus, our integration score was an agreed-upon score on the level of involvement among agencies. For example, if there were 80% of agencies whose group-perceived scores on Agency X were same as their group-expected scores, then the corresponding degree of integration for Agency X would be 80%. Our integration framework measured the agreement from four perspectives shown in Figure 3, including the agreement between: the group-perceived and group-expected involvements (P1), the self-perceived and group-expected involvements (P2), the group-perceived and self-expected involvements (P3), and the self-perceived and self-expected involvements (P4).

### Calculating the global integration score

**Figure 4.**
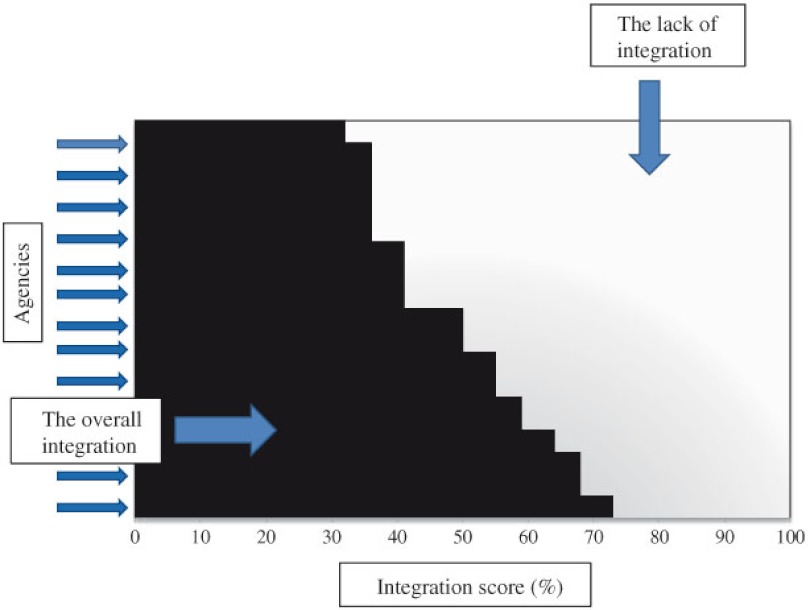
An example of the global integration.

We estimated the global integration score of a network by the average integration score. As shown in Figure 4, the graded area represented the global integration score of a network based on scores from all the network agencies. The blank area on the diagram indicates the gap in the degree of global network integration. The total area is the sum of the graded and the blank areas of the diagram which equals 1 (i.e., 100% integration). Essentially, the graded area represents the average integration score. We calculated corresponding global integration score with 95% confidence interval (CI) as the graded area based on P1, P2, P3, and P4 integration scores.

### Assessing the sensitivity of global integration score

We conducted a sensitivity analysis by comparing with other methods for estimating the global integration score: the weighted-average method and the bootstrap method. In the weighted-average method, we attached a different weight (*w*) to each agency based on the variance of its integration score. This method adjusted for the precision of an estimated integration score. An agency with a more precise integration score contributed more to the global score for the whole network. For the bootstrapping method, we used three different bootstrap procedures: the standard, the balanced, and the Bayesian procedures. Bootstrap is a common resampling method for improving estimation and confidence intervals of an unknown parameter [27–29]. Different procedure requires a different resampling algorithm and thus, estimates the sampling error differently. The standard bootstrap can produce a bias-corrected estimate [29] that largely reduces the potential bias arising in estimation. The balanced bootstrap is similar to the standard procedure but bootstrap samples are balanced. Compared with the standard bootstrap, the balanced bootstrap generally improves the efficiency of simulation [30]. The Bayesian bootstrap uses a different algorithm and approximates a posterior distribution of the global score instead of a sampling distribution [31]. For both the standard and balanced bootstrap procedures, we computed the 95% bias-corrected and accelerated CI that adjusted both bias and skewness in bootstrap sampling [28]. For the Bayesian bootstrap procedure, we computed the 95% credibility interval (CrI) instead. The details of calculations and procedures were provided in the Appendix. We performed all statistical analyses in the software package R version 2.12.1.

## Results

### The integration scores for agencies of CTN

We summarized the integration scores for CTN agencies in Table 1. For confidentiality, we kept agencies anonymous and labeled them by Arabic numbers. The response rate was 89% in Simcoe and 64% in York. All agencies (i.e., both respondents and non-respondents) received a P1 score. However, only respondents received P2, P3, and P4 scores because those calculations required the agencies’ ratings on other agencies.

In Simcoe, P1, P2, P3, and P4 integration scores varied from 25% to 82%, 4% to 83%, 13% to 83%, and 4% to 96%, respectively. In York, P1, P2, P3, and P4 integration scores varied from 27% to 73%, 27% to 81%, 9% to 82%, and 23% to 97%, respectively.

### The global integration scores for the CTN

Global integration scores of CTN were summarized in Table 2. In Simcoe, P1, P2, P3, and P4 global integration scores with 95% CI were 44% (39%, 49%), 43% (36%, 51%), 43% (35%, 52%), and 44% (32%, 55%), respectively. In York, they were 52% (48%, 56%), 54% (48%, 61%), 54% (45%, 63%), and 52% (43%, 61%), respectively. The global integration of CTN in York was generally higher than that in Simcoe.

### Assessing the global integration score

**Figure 5.**
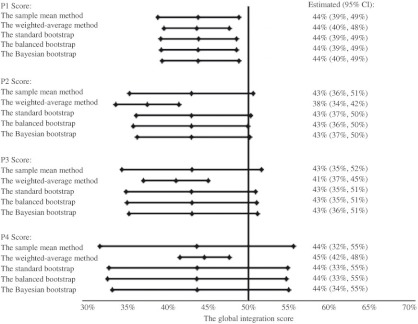
Global integration scores of CTN Simcoe.

**Figure 6.**
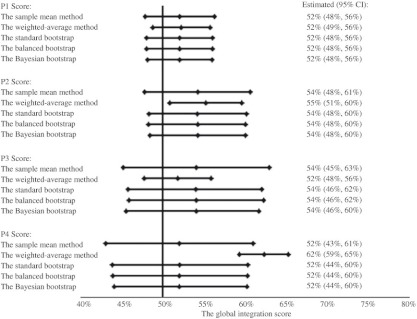
Global integration scores of CTN York.

The global integration scores calculated by different approaches were similar. We performed all bootstrap procedures by simulating 500, 1000, 5000, 10,000, and 40,000 bootstrap samples. Although, increasing the number of simulations reduced the random sampling error caused by the bootstrap procedure itself, the results only differed in the third decimal place. Thus, we only reported the results by simulating 40,000 bootstrap samples. The weighted-average approach provided a slightly different estimate in some cases and the narrowest 95% CI (Figures 5 and 6). Other researchers also reported a narrower confidence interval when using a weighted approach [32]. Still, the 95% CIs covered all scores by different methods. The only exception was the P4 global score measuring the overall agreement between self-perceived and self-expected involvements in York, where the weighted-average method provided a significantly larger estimate than other methods. The global scores estimated by different bootstrap procedures were identical to the standard one. This showed that the average integration score was a simple and reliable estimate of the global score. The findings were consistent with the fact that a sample mean was an unbiased estimate of the population mean. The 95% CIs in bootstrap methods were slightly more precise. Overall, the sample mean was a robust estimate of the global integration score.

## Discussion

**Figure 7.**
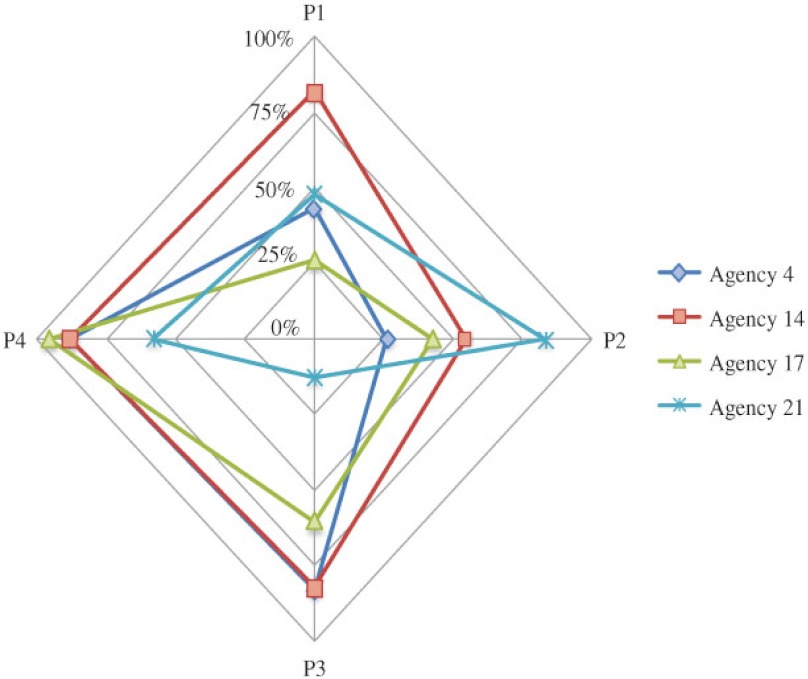
The spider plot for comparing the 4 integration scores for agencies in Simcoe.

**Figure 8.**
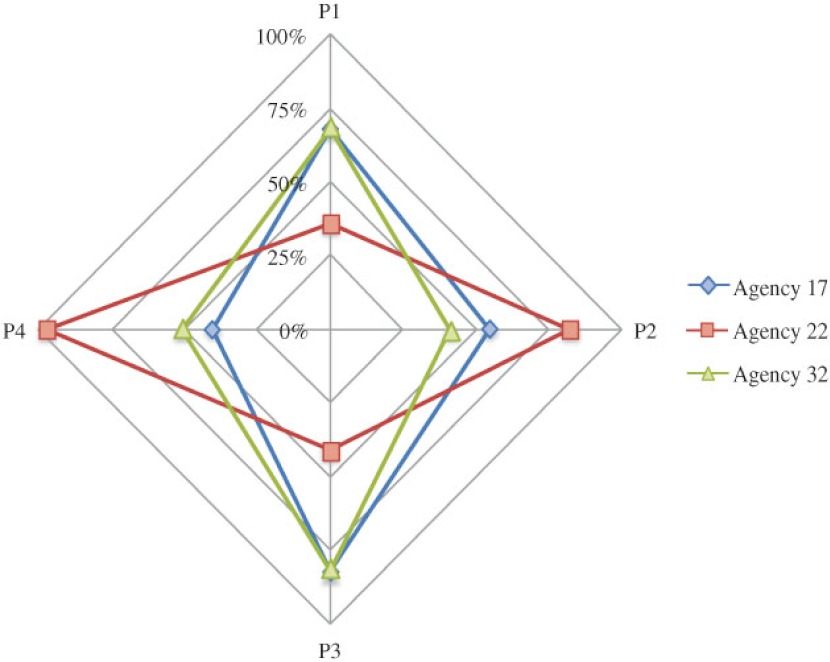
The spider plot for comparing the 4 integration scores for agencies in York.

We have developed a method for quantifying the degree of integration for agencies in an integrated service network. Using this method, managers could identify the current gap in service integration. We applied the method in measuring the CTN agencies. Non-respondent agencies had lower P1 integration score, which indicated a poorer integration as perceived and expected by the group. For some agencies, their scores (i.e., P1, P2, P3, and P4 scores) varied largely across different perspectives of integration. As shown in Figures 7 and 8, the spider plots were helpful for examining the gap of integration by different views. When an agency had 100% integration, the plot would show a complete ‘diamond’. Any defect on the ‘diamond’ would indicate imperfect integration from some perspective. For example, in Simcoe, Agency 4 had much lower P1 and P2 scores than its other scores: below 45% vs. above 80%. This suggested that the level of involvement perceived by all agencies including Agency 4 itself did not meet other agencies’ expectation. This was an indication that the group might have a wrong expectation on the level of involvement required for Agency 4. Agency 14 had a much lower P2 score than other scores: 55% vs. above 80%. The level of involvement perceived by Agency 14 met its own expectation but not others. This was an indication that Agency 14 might have an improper perception on the level of involvement it was contributing to the group. Agencies 17 had much lower P1, P2, and P3 scores than its P4 score: below 62% vs. above 85%. This showed that the level of involvement perceived by Agency 17 only met its own expectation but not others, and was different from others’ perception too. Agency 21 had much lower P1, P3, and P4 scores than its P2 score: below 59% vs. above 82%. Although Agency 21’s perception on its current involvement met others’ expectation, it was different from what others had perceived. Our findings helped CTN managers to diagnose deeper problems of integration and potential barriers in integrating multiple services. By measuring the service integration over time, we could also evaluate the improvement in agency working relationships and promote further dialogue in achieving better integration.

Our measurement had some limitations. First, we only focused on measuring the degree of collaborative involvement in service integration. There were other types of integration [33], e.g., the functional integration, which we did not measure. Second, the measurement only captured the integration among the planning group. We acknowledged that integration achieved at the planning table did not always reflect the degree of integration in real practice, for example, among the frontline teams of workers. Third, there was potential respondent bias because filling out the measure by representative(s) is subject to proxy reporting bias. Representatives might not give the same information that others from the same agency would give. Halo effect and end-aversion were two other potential sources of bias in our results [34]. Finally, we were not able to evaluate the impact of non-respondents on the results.

Integration models generally require some formal mechanism, such as networks or committees of local agencies, to plan, organize, and deliver multiple services together. There are many barriers to integration because current health, educational, social, rehabilitative services, etc. are funded independently. Relationship, politics, communication, process, structure, and conflict are common problems for the failure of integration [35–38]. The gains from integration are often difficult to sustain and we need a tool to measure them. Despite the limitations, our method provides a valid way to conceptualize and quantify the service integration among agencies. During the time of measurement, CTN was undergoing initial planning to link resources and support, organize services, and create a new governance model. This partly explained the low degree of integration that CTN agencies had achieved. By quantifying the state of integration, our measurement helped CTN agencies visualize their agreement in the process of integration and generated important discussions for their next stage of planning. The unique value of our tool is that it provides a relative score on the degree to which the actual involvement agrees with the expected involvement. In this regard, our method greatly extends the HSIM by bridging the perceived and the expected integrations. Compared with the Ahgren and Axelsson’s method [23], our approach does not adopt an assumption that higher levels of integration are better. Another difference was that the Ahgren and Axelsson’s method uses specific criteria to define levels of integration (e.g., referrals, guidelines, chains of care, network managers, and pooled resources). Although this might be a more objective approach, the measure could not distinguish the differences in the degree of involvement that should be present in a well-functioning network. In addition, our method produces simple and reliable global integration scores that quantify the integration of an entire network as a whole, which is hardly addressed in the current literature. Our method possesses a good content relevance and coverage on measuring integration by including: the spectrum of services, the number of providers, the score of integration, and the perspective of agencies. Our method can also differentiate the degree of integration between similar networks, as shown in case of the CTN Simcoe and York.

We have used the Partnership Self-Assessment Tool [39, 40] to examine the association between our integration scores and components of a collaborative process—synergy, leadership, efficiency, administration and management, resources, decision-making, benefits, drawbacks, and satisfaction. Our results showed that synergy was strongly associated with integration [41]. Other components including leadership, administration, decision-making, and satisfaction were also associated with integration. The findings demonstrated some extent of convergent validity because our integration measure was related to other variables of the same construct to which it should be related [34]. In an on-going analysis, we are examining the linkage between the degree of integration and network capacity that includes the average wait-time and the caseload. For future studies, we can apply the measurement in other similar service networks or in the same network for continuous evaluation. By repeating the measurement, investigators can determine integration patterns over time and examine the connection between integration and network outcomes longitudinally. We have found a potential influence of provider team integration on the quality of life of children with complex needs [42]. Future studies can also examine the similar relationship between different patient outcomes and the degree of integration using our measure.

## Concluding remarks

In this paper, we introduce a method for measuring the degree of integration for agencies in an integrated service network. Using the method, we measured the integration of the CTN agencies in Simcoe and York. In CTN Simcoe, agencies’ P1, P2, P3, and P4 integration scores varied from 25% to 82%, 4% to 83%, 13% to 83%, and 4% to 96%, respectively. In CTN York, agencies’ P1, P2, P3, and P4 integration scores varied from 27% to 73%, 27% to 81%, 9% to 82%, and 23% to 97%, respectively. Most agencies had a score of <65%, a relatively low level of integration. The results revealed existing problems in integrating CTN services. As measured by the agreement between every other agency’s perception and expectation, the overall integration of CTN in Simcoe and York was 44% (95% CI: 39%–49%) and 52% (95% CI: 48%–56%), respectively. The sensitivity analysis showed that the average integration score was a reliable and robust estimate of the global integration score. The measurement provided timely information for decision-making and improving the integration of CTN. The key implication is that every integrated service network needs a valid measurement to evaluate whether or not the collaborative process has been implemented as planned. Measuring the service integration should be the first step in this evaluation. However, quantitative methods available for measuring integration have been scarce in the literature. Our method greatly extends the existing measures of integration by quantifying the agreement between agencies’ perceived and expected levels of involvement. Our approach is unique such that we quantify the integration from four different perspectives to identify deeper problems in integrating multiple services provided by autonomous agencies. We showed that the proposed method possessed a good extent of validity and could be applied in measuring other integrations in a similar setting.

## Authors’ contributions

CY conceived the study, designed the integration scores, performed all analyses, interpreted the results, and drafted and revised the manuscript. GB advised on important intellectual content and revised the manuscript. VSG revised the manuscript and contributed to the interpretation of results. JB revised the manuscript and contributed to the statistical analysis of the study. LT contributed to the statistical design, interpretation of results and revision of the manuscript. All authors have read and approved the final manuscript.

## Acknowledgements

We thank Health and Social Service Utilization Research Unit (HSSURU) at McMaster University for providing the data. Chenglin Ye is supported in part by funding from the Father Sean O’Sullivan Research Center (FSORC) Studentship award, the Canadian Institute of Health Research (CIHR) Training award in Bridging Scientific Domains for Drug Safety and Effectiveness, and the Canadian Network and Centre for Trials Internationally (CANNeCTIN) programme.

## Reviewers

**Reynaldo Holder**, MD, Regional Advisor—Hospitals and Integrated Health Care Delivery Systems, Area of Health Systems based on Primary Health Care, PAHO/WHO, Washington DC, USA.

**Nick Goodwin**, Senior Fellow, The King’s Fund, London, UK.

**Daniel Salhani**, PhD, Associate Professor, School of Social Work, University of British Columbia, Okanagan Campus, Canada.

## Figures and Tables

**Table 1.**
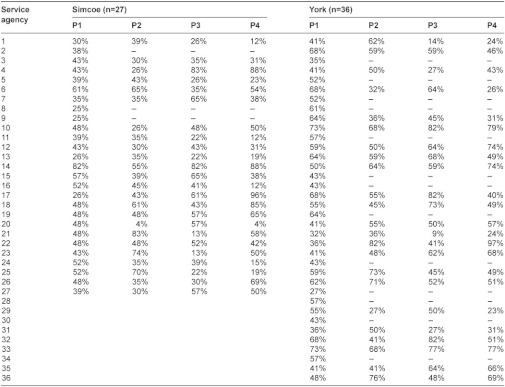
Integration scores for the agencies of the Children’s Treatment Network (CTN)

Percentages were rounded up to integer; the agencies were listed in a consecutive order and the same number ***did not*** refer to the same agency; CI=confidence interval.

P1=the degree of agreement between an agency’s involvement perceived by others and its involvement expected by others.

P2=the degree of agreement between an agency’s self-perceived involvement and its involvement expected by others.

P3=the degree of agreement between an agency’s involvement perceived by others and the involvement expected itself.

P4=the degree of agreement between an agency’s self-perceived involvement and the involvement expected itself.

**Table 2.**
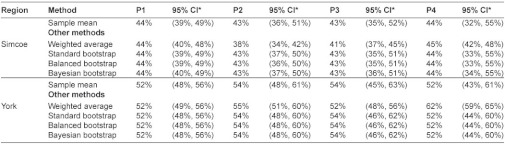
Global integration scores estimated by different methods

*CI=confidence interval, a credibility interval was calculated instead in the Bayesian bootstrap method; n=number of respondents; bootstrap estimates were obtained by simulating 40,000 bootstrap samples.

P1=the degree of agreement between an agency’s involvement perceived by others and its involvement expected by others.

P2=the degree of agreement between an agency’s self-perceived involvement and its involvement expected by others.

P3=the degree of agreement between an agency’s involvement perceived by others and the involvement expected itself.

P4=the degree of agreement between an agency’s self-perceived involvement and the involvement expected itself!

## APPENDIX—Statistical Methods

**Calculating the integration scores**

The small letter *n* denotes the number of respondents. Since an agency does not rate it itself, the number of respondents is the total number of agencies less 1 when there is no non-respondent. The integration score  is calculated by

where *I*_*i*_ is an indicator that returns 1 if the perceived involvement is equal to the expected involvement and 0 otherwise for the *i*th pair of ratings. Current perception of integration can be either self- or group-perceived. Similarly, expectation can be either self- or group-expected. The variance of integration score is calculated by

where  is the P1, P2, P3 or P4 score.

**Calculating the global integration score**

**The standard method**

The capital letter *N* denotes the total number of agencies within a network. The average integration score estimates the global integration , given by

where  is the estimated P1, P2, P3, or P4 score of the *i_th_* agency.

The 95% confidence interval (CI) of a global integration score is calculated by

where *s* is the sample standard deviation of the integration scores.

**The weighted-average method**

The weighted global integration score is calculated by

where  is the integration score of the *i_th_* agency and *w*_*i*_ is the inverse of variance of .

The 95% CI of the weighted global integration score is calculated by.

**The bootstrap method**

In the standard bootstrap, we randomly draw an integration score from all agencies with replacement repeatedly to generate a bootstrap sample. We repeat that procedure to generate a large number of *k* bootstrap samples. The choice of *k* often depends on the computational power. In the balanced bootstrap, every integration score appears the same number of times among bootstrap samples. In other words, each original integration score is selected *k* times and allocated randomly in the *k* bootstrap samples. We achieve that by creating *k* replicates of original sample and then randomly sampling without replacement among those replicates to create *k* equal bootstrap samples. We calculate the mean of each bootstrap sample by

where  is the average integration score for the *j_th_* bootstrap sample and s are the integration scores selected in that bootstrap sample.

When *k* is large enough (usually over 1000), aggregating the means of all bootstrap samples constitutes a sampling distribution of the global score. The bias in estimating the global score can be then approximated by

where _*b*_ is the expected global integration score from bootstrap samples and  is the mean of the original sample.

The bias-adjusted bootstrap estimate of the global integration score is calculated by

The 95% bias-corrected and accelerated (BCa) confidence interval for the global integration score is (*m*_1_, *m*_2_) where

The Ф(·) is the standard normal cumulative function; *â* is the acceleration; and  is the bias-correction. The method of calculating *â* and  empirically is provided in [28].

In the Bayesian bootstrap, each bootstrap sample generates a posterior probability for each integration score in the original sample. We draw *N*–1 uniform random numbers between 0 and 1, order them to be *c*_*1*_, *c*_*2*_, …, *c*_*N*_, and let *c*_0_=0 and *c*_1_=1. We then calculate the gaps *g*_*i*_=*c*_*i*_–*c*_*i*__-1_, *i*=1,…,*N* and attach *g*=(*g*_1_,…,*g_N_*) as the vector of probabilities to the original integration scores  for that bootstrap sample. We repeat that procedure to create *k* bootstrap samples. Details of the procedure can be found in [31]. The posterior estimate of the average integration scores in each bootstrap sample is calculated by,

The Bayesian bootstrap (BB) estimate of the global integration score can be calculated by the average of all posterior estimates from bootstrap samples, i.e.,

The 95% credibility interval can be obtained from the empirical distribution of all posterior estimates ’s.
